# Evaluación del daño oxidativo y por metilación del ADN de pintores expuestos ocupacionalmente a solventes orgánicos y pintura

**DOI:** 10.7705/biomedica.4289

**Published:** 2019-09-01

**Authors:** Elizabeth Londoño-Velasco, Fabián Martínez-Perafán, Silvio Carvajal, Felipe García-Vallejo, Luz Stella Hoyos-Giraldo

**Affiliations:** 1 Grupo de Investigación en Toxicología Genética y Citogenética, Departamento de Biología, Facultad de Ciencias Naturales Exactas y de la Educación, Universidad del Cauca, Popayán, Colombia Universidad del Cauca Departamento de Biología Facultad de Ciencias Naturales Exactas y de la Educación Universidad del Cauca Popayán Colombia; 2 Departamento de Ciencias Básicas de la Salud, Facultad de Ciencias de la Salud, Pontificia Universidad Javeriana, Cali, Colombia Pontificia Universidad Javeriana Departamento de Ciencias Básicas de la Salud Facultad de Ciencias de la Salud Pontificia Universidad Javeriana Cali Colombia; 3 Instituto de Fisiopatología y Bioquímica Clínica, Departamento de Bioquímica Clínica, Facultad de Farmacia y Bioquímica, Universidad de Buenos Aires, Ciudad Autónoma de Buenos Aires, Argentina Universidad de Buenos Aires Departamento de Bioquímica Clínica Facultad de Farmacia y Bioquímica Universidad de Buenos Aires Ciudad Autónoma de Buenos Aires Argentina; 4 Laboratorio de Biología Molecular y Patogénesis, Departamento de Ciencias Fisiológicas, Facultad de Salud, Universidad del Valle, Cali, Colombia Universidad del Valle Departamento de Ciencias Fisiológicas Facultad de Salud Universidad del Valle Cali Colombia

**Keywords:** exposición ocupacional, daño del ADN, ensayo cometa, ADN-formamidopirimidina glicosilasa, N-glucosil hidrolasas, Occupational exposure, DNA damage, comet assay, DNA-formamidopyrimidine glycosylase, N-glycosyl hydrolases

## Abstract

**Introducción.:**

La exposición a solventes orgánicos y pinturas se ha asociado con efectos genotóxicos y mayor riesgo de neoplasias. Sin embargo, aún no se ha caracterizado bien el tipo de daño que esta exposición induce en el ADN humano, ni los mecanismos por los cuales se genera. Uno de los grupos con mayor exposición a dichos solventes y pinturas son los pintores de automóviles del sector informal que trabajan sin adecuadas prácticas de seguridad ocupacional.

**Objetivo.:**

Determinar el daño oxidativo y por metilación del ADN de linfocitos de pintores de automóviles expuestos a solventes orgánicos y pinturas.

**Materiales y métodos.:**

Se analizaron linfocitos aislados de sangre periférica de 62 pintores y 62 sujetos no expuestos mediante el ensayo cometa de gran eficiencia acoplado a las enzimas Fpg y AlkA. Las categorías de daño en el ADN evaluadas fueron el daño basal (sin enzimas), el daño oxidativo y el daño por metilación, y el parámetro de medición, el porcentaje de ADN en la cola.

**Resultados.:**

El porcentaje de ADN en la cola fue mayor en el grupo expuesto con respecto al no expuesto (p<0,05). En el grupo expuesto, dicho porcentaje fue mayor en la categoría de daño oxidativo comparado con la del basal (16,50 *Vs.* 12,87; p<0,001), en tanto que en el daño por metilación no se encontraron diferencias significativas (14,00 *Vs.* 12,87; p>0,05).

**Conclusión.:**

La exposición a solventes orgánicos y pinturas se asoció con el aumento de las lesiones oxidativas del ADN de los linfocitos de pintores de automóviles, tales como la producción de 8-oxo-2’-desoxiguanosina (8-oxodG) y otros productos formamidopirimidina, los cuales se consideran considerablemente mutagénicos.

## Abstract (en)

**Introduction:**

The exposure to organic solvents and paints has been associated with genotoxicity and a greater risk of neoplasms. However, the type of DNA damage induced in humans by the exposure to these compounds, which would help explain the mechanisms of their genotoxicity, is still not fully characterized. Due to inadequate practices of occupational safety, car painters in the informal sector are a highly exposed group to organic solvents and paints.

**Objective:**

To identify the oxidative and methylating damage in the DNA of lymphocytes of car painters exposed to organic solvents and paints.

**Materials and methods:**

Isolated peripheral blood lymphocytes from 62 painters and 62 unexposed subjects were analyzed by the modified high-throughput comet assay with the Fpg and AlkA enzymes. The categories used for the evaluation of the DNA damage were basal damage (without enzymes), oxidative and methylating damage. The measurement parameter used to establish the damage was the percentage of DNA in the tail.

**Results:**

The percentage of DNA in the tail was higher in the exposed group compared to the unexposed group (p<0.05).

In the exposed group, this percentage was higher in the oxidative damage category than the baseline (16.50 vs. 12.87; p<0.001), whereas methylating damage did not show significant differences (14.00 vs. 12.87; p>0.05).

**Conclusion:**

In this study, exposure to organic solvents and paints was associated with an increase in oxidative lesions in the DNA of car painters’ lymphocytes, such as the production of 8-oxodG and other formamidopyrimidine products which are considered highly mutagenic.

En las últimas décadas, las estrategias para abordar la problemática de la exposición a xenobióticos han cambiado. Actualmente es de gran interés para la comunidad científica evaluar, no solo las fuentes y niveles de exposición ambiental, sino también los efectos biológicos tempranos en el material genético, para detectar y alertar sobre los factores de riesgo de los problemas de salud asociados con la exposición ocupacional o ambiental a tales xenobióticos.

Los pintores de automóviles son una población expuesta a metales pesados -plomo, cromo y aluminio, entre otros-, a pigmentos residuales de monómeros de plástico contenidos en las pinturas y a mezclas complejas de solventes orgánicos (hidrocarburos aromáticos y alifáticos, cetonas, alcoholes y ésteres, entre otros) ^1^^-^^3^.

El diluyente (*thinner*) es la mezcla compleja de solventes orgánicos más usada entre los pintores de automóviles; en estudios *in vitro* se ha demostrado que este compuesto tiene efectos genotóxicos en linfocitos humanos ^4^.

En otros estudios *in vitro* e *in vivo* se ha demostrado que mediante el proceso de biotransformación de los solventes orgánicos, se generan especies reactivas de oxígeno y radicales libres que afectan significativamente a los lípidos, las proteínas y el ADN ^5^^,^^6^.

En los estudios de biomonitoreo que emplean biomarcadores de efecto temprano, como la frecuencia de alteraciones cromosómicas, los micronúcleos y el daño del ADN determinado mediante el ensayo cometa, también se ha demostrado el potencial genotóxico de los solventes orgánicos y, en consecuencia, el riesgo de desarrollar cáncer por la exposición ocupacional crónica a ellos ^7^^-^^11^.

Sin embargo, son muy pocas las investigaciones en las que se ha caracterizado el tipo de daño del ADN (oxidativo o por metilación) de células humanas de individuos expuestos a estos compuestos, lo que permitiría dilucidar el mecanismo de acción de los solventes orgánicos y de las pinturas sobre el material genético, además de evidenciar posibles deficiencias en los mecanismos de reparación del ADN, lo cual constituye un factor de riesgo para el desarrollo de enfermedades asociadas con la inestabilidad genética.

El ensayo cometa es una prueba citogenética molecular cuya sensibilidad permite detectar pequeños daños genéticos (roturas de cadena, sitios lábiles al álcali y sitios de reparación por escisión incompleta de bases) que se presentan como daño basal en células normales o como resultado de la exposición a pequeñas dosis de agentes genotóxicos ^12^^,^^13^.

Para incrementar la sensibilidad y la especificidad de esta prueba, se han incorporado tratamientos enzimáticos con glucosilasas (enzimas de la reparación del ADN), las cuales convierten las lesiones específicas en roturas de cadena que incrementan la intensidad de la cola del cometa (indicador de un aumento de daño en el ADN) en comparación con los controles no tratados con tales enzimas, y permiten estimar la frecuencia de este tipo de lesión en el ADN.

Algunas de las enzimas utilizadas en el ensayo cometa, son la formamidopirimidina ADN glucosilasa (Fpg), que remueve bases oxidadas (7,8-dihidro-8-oxo-2´deoxiguanina 8-oxoGua) u otros productos formamidopirimidina (4,6-diamino-5-formamidopirimidina FapydAde y 2,6-diamino-4-hidroxi-5-formamidopirimidina FapydGua), así como la 3-metiladenina-ADN glucosilasa II (AlkA), la cual remueve bases metiladas (3-metiladenina 3-MeAde y 7-metilguanina 7-MeGua) y genera sitios sin purina que, posteriormente, son transformados en roturas de cadena cuando el ADN es sometido a un pH alcalino durante la electroforesis ^14^^,^^15^.

En este contexto, el objetivo del presente estudio fue estimar el daño oxidativo y por metilación del ADN de los linfocitos de pintores de automóviles expuestos a solventes orgánicos y a pinturas mediante el ensayo cometa de gran eficiencia con la inclusión de las enzimas Fpg y AlkA, respectivamente.

## Materiales y métodos

Grupos de estudio

Se hizo un estudio de corte transversal y analítico para comparar grupos expuestos y no expuestos. El muestreo fue no probabilístico (por conveniencia) e incluyó a hombres saludables en un rango de edad entre los 18 y los 55 años, todos residentes en el departamento del Cauca, Colombia. Se excluyeron del estudio a los fumadores, a quienes manifestaron padecer infecciones agudas virales o bacterianas en el momento de la toma de la muestra, a quienes tenían antecedentes de cáncer y a quienes habían sido sometidos a quimioterapia o algún tipo de radiación con fines terapéuticos o diagnósticos durante los tres meses previos a la toma de la muestra de sangre.

Con base en estos criterios de inclusión y exclusión, se seleccionaron 62 pintores en talleres de latonería y pintura de automóviles del sector informal y a 62 individuos no expuestos ocupacionalmente a solventes y pinturas. Los individuos del grupo expuesto habían estado laborando como pintores por cinco años o más. Los individuos de ambos grupos se emparejaron por edad (±2 años) y estrato socieconómico.

El estudio fue evaluado y avalado por los comités de ética de la Universidad del Cauca y de la Universidad del Valle siguiendo los lineamientos de las directrices de la Declaración de Helsinki ^16^, el Consejo de Organizaciones Internacionales de las Ciencias Médicas (CIOMS), la Organización Mundial de la Salud (OMS) ^17^ y la Resolución 8430 de 1993 de Colombia ^18^.

Todos los participantes del estudio fueron informados sobre los objetivos, la metodología y los riesgos de la investigación antes de obtener su consentimiento informado. Asimismo, se elaboró la anamnesis con sus datos demográficos, antecedentes ocupacionales, estado de salud y estilo de vida.

Muestras de sangre y aislamiento de linfocitos

Las muestras de sangre se tomaron a lo largo de tres meses (junio a agosto), en horas de la mañana (entre 9:00 am y 12:00 m) del tercer día de una semana de actividad laboral. De cada participante se obtuvieron 5 ml de sangre periférica por venopunción en tubos vacutainer con ácido etilendiaminotetraacético disódico (EDTA). Los linfocitos fueron aislados con Ficoll-Histopaque™ (1077-1 Sigma), y el sedimento celular se suspendió de nuevo en 1,5 ml de medio RPMI (*Roswell Park Memorial Institute*) 1640™ (R-8758 Sigma).

Controles positivo y negativo

El correcto desarrollo de la metodología del ensayo y la eficiencia de cada corrida electroforética se comprobaron usando controles internos (positivo y negativo). Para establecer dichos controles, se hicieron cultivos primarios y el tratamiento *in vitro* de linfocitos aislados de sangre periférica de un donador voluntario de 23 años, saludable y no fumador.

Se cultivaron aproximadamente 3,0 x 104 linfocitos por pozo con medio RPMI 1640 (pH 7,2), con suplemento de L-glutamina, antibiótico, fitohemaglutinina y suero bovino fetal en microplatos de 96 pozos durante 24 horas a 37 °C y 5 % de CO2. Para el control interno positivo, se usaron linfocitos tratados con 40 µM de peróxido de hidrógeno y H2O2 (CAS 7722-84-1™, Sigma, 30 % v/v) durante 30 minutos, y 2,26 mM de metanosulfonato de etilo (EMS) (CAS 62-50-0™, Sigma, >99 %) durante 2 horas a 37 °C. Como control negativo se utilizaron linfocitos suspendidos en solución tampón de fosfato salino (PBS, P-3813-10™, Sigma).

Viabilidad celular

Los linfocitos de las muestras de la población de estudio y de los controles internos (positivo y negativo), se contaron y se analizaron para determinar la viabilidad celular mediante la prueba de exclusión con el colorante vital azul de tripano (T-6146™, Sigma) al 0,1 % en PBS. Los linfocitos aislados de los participantes presentaron una viabilidad mayor de 96 % y la de los cultivados como controles internos estuvo entre 80 y 90 % (no se incluyen los datos).

Ensayo cometa de gran eficiencia y digestión enzimática

Para el ensayo cometa (electroforesis de células individuales), se empleó el método alcalino estándar de Singh, *et al.*^19^ con algunas modificaciones para la versión de gran eficiencia ^20^^,^^21^ y la inclusión de las enzimas glucosilasas Fpg (ADN-formamidopirimidina glucosilasa, Cat. M0240L™, New England Bio-Labs) y AlkA (ADN-alquiladenina glucosilasa humana, Cat. M0313™, New England Bio-Labs) ^12^^,^^14^. Con dichas modificaciones se buscaba simplificar el proceso incrementando el número de muestras que se procesaban en cada montaje experimental sin alterar la capacidad de detección del daño del ADN en cada muestra. Por ello, estas modificaciones fueron validadas (calibración) en el Laboratorio de Toxicología Genética y Citogenética de la Universidad del Cauca antes de aplicarlas en los montajes experimentales concretos (no se incluyen los datos).

**Figura 1:**
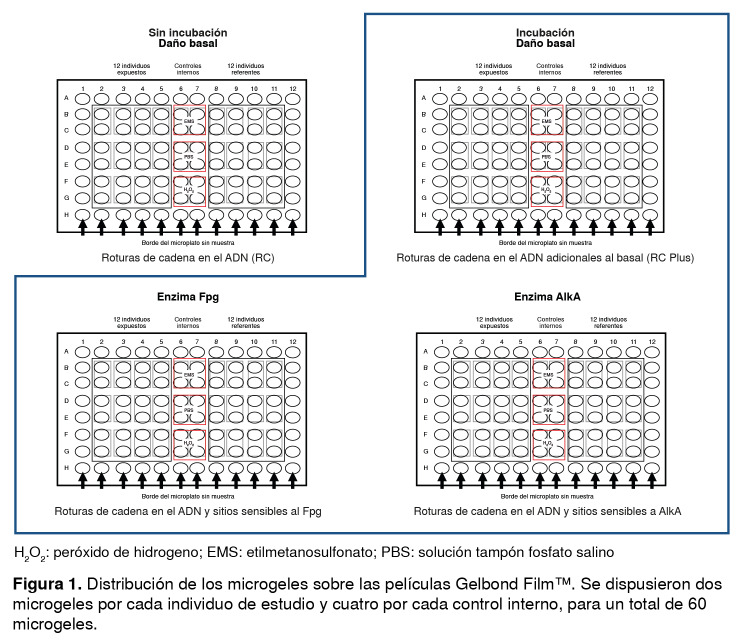
Distribución de los microgeles sobre las películas Gelbond Film™. Se dispusieron dos microgeles por cada individuo de estudio y cuatro por cada control interno, para un total de 60 microgeles.

Para obtener los microgeles, se embebieron aproximadamente 10.000 linfocitos por individuo en 90 µl de agarosa de bajo punto de fusión (A-9414™, Sigma) al 0,5 %. De esta suspensión celular se tomaron 5 µl que fueron depositados sobre una película Gelbond Film™ (Lonza Copenhagen ApS, Denmark, 54727). En cada Gelbond Film™ de 16 x 11 cm se depositaron muestras duplicadas de individuos expuestos y no expuestos (dos microgeles por individuo), al igual que las muestras de los controles internos positivo y negativo. Para el ensayo con incubación enzimática, se prepararon cuatro Gelbond Film™ idénticas por montaje experimental, las cuales se etiquetaron como ‘daño basal’, ‘solución tampón de reacción’, ‘Fpg’ y ‘AlkA’ (figura 1).

Después de la solidificación de los microgeles, las cuatro Gelbond Film™ se sumergieron en una solución de lisis (cloruro de sodio, NaCI 2,5 M, S-9625™, Sigma), 100 mM de Na2EDTA (E-5134™, Sigma), Tris 10 mM (T-3253™, Sigma); tritón X-100 (K-0320™, Sigma) al 1 % (v/v), y dimetilsulfóxido (DMSO) (D-8779™, Sigma) al 10 % (v/v) y pH 10, y se mantuvieron en la oscuridad durante toda la noche a 4 °C.

La película Gelbond Film™ sin tratamiento enzimático ni incubación se etiquetó como daño basal y sirvió como valor de referencia correspondiente a la totalidad de daños del ADN, incluidas las roturas de cadena simples o dobles y las originadas por sitios lábiles al álcali o sitios de reparación incompleta, sin hacer distinción entre ellas, en tanto que las Gelbond Film™ etiquetadas como ‘solución tampón de reacción’, ‘Fpg’ y ‘AlkA’ se sometieron a incubación y tratamiento enzimático para revelar las roturas de cadena y las lesiones específicas en el ADN (8-oxodG, FapydAde, FapydGua, 3-meA y 7-meG).

Antes de la incubación, las tres Gelbond Film™ se lavaron con solución tampón de reacción enzimática (HEPES 70 mM, 83264™, Sigma), KCl 0,1 M (P9541™, Sigma), 2 mM de Na2EDTA (E-5134™, Sigma) y 0,26 mg/ml de SBA (Fermenta™s), y pH de 7,6. Posteriormente, se aplicaron por goteo alícuotas de 50 µl de la solución tampón de reacción o de las enzimas ‘glucosilasas’ Fpg y AlkA con una concentración de 1 µg/ml sobre cada uno de los microgeles en las Gelbond Film según su etiqueta y se incubaron durante 30 minutos a 37 °C.

Para la desnaturalización y el ‘desenrollamiento’ del ADN, las cuatro Gelbond Film™ se sumergieron en solución tampón alcalina (NaOH 10 N, S-8045™, Sigma; 200 Mm de Na2EDTA, E-5134™, Sigma, pH 13,3) durante 40 minutos y, luego, se sometieron a electroforesis durante 30 minutos a 28 V y 300 mA (0,9 V/cm).

**Figura 2:**
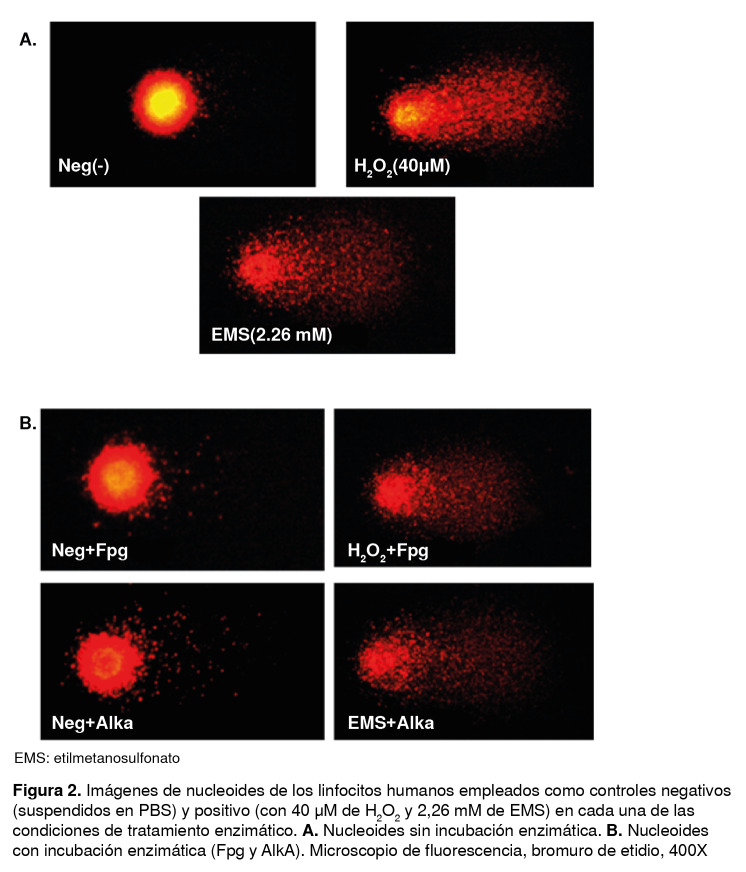
Imágenes de nucleoides de los linfocitos humanos empleados como controles negativos (suspendidos en PBS) y positivo (con 40 µM de H2O2 y 2,26 mM de EMS) en cada una de las condiciones de tratamiento enzimático. **A.** Nucleoides sin incubación enzimática. **B.** Nucleoides con incubación enzimática (Fpg y AlkA). Microscopio de fluorescencia, bromuro de etidio, 400X

Los microgeles se lavaron con una solución tampón de neutralización (Tris 0,4 M, T-3253™, Sigma, pH 7,5) para retirar la solución alcalina y, posteriormente, se deshidrataron con etanol absoluto frío. Las condiciones de tratamiento enzimático y de electroforesis se consideraron óptimas y válidas solo si los controles internos positivo y negativo daban los resultados esperados después de la electroforesis (figura 2).

Para el registro de las pruebas cometa, los microgeles se colorearon con bromuro de etidio (20 µg/ml, E-7637™, Sigma). Se hizo un recuento sistemático aleatorio de 50 nucleoides por cada microgel (100 nucleoides por individuo), con una amplificación de 400X en un microscopio de fluorescencia (Nikon Eclipse E400™). Las imágenes se analizaron con el programa Komet 6™ (Kinetic Imaging Ltd., Liverpool, Reino Unido). El daño en el ADN de cada participante se determinó mediante el porcentaje de ADN en la cola y aquellos nucleoides indicadores de muerte celular (*clouds*) se excluyeron del registro.

Análisis estadístico de los datos

Para obtener los valores del daño oxidativo o del daño neto por metilación se calculó la diferencia entre el valor de porcentaje de ADN en la cola obtenido tras la digestión enzimática (con Fpg o AlkA) y el valor adicional en la ruptura de cadenas. Este valor adicional se determinó mediante la diferencia entre los valores del porcentaje de ADN en la cola obtenido después de la incubación con solución tampón de reacción y aquel obtenido sin incubación enzimática (daño basal).

Los datos se analizaron mediante la prueba de Kolmogorov-Smirnov para determinar la normalidad y la de Levene para la homogeneidad de las varianzas. Las características demográficas se analizaron mediante la prueba t de Student y la de ji al cuadrado (χ2). Para determinar las diferencias entre las categorías de daño del ADN y los grupos de estudio, se utilizaron las pruebas de Kruskal-Wallis y la U de Mann-Whitney. La categoría de daño basal se consideró como el valor de referencia para comparar la frecuencia del daño oxidativo o del daño neto por metilación.

La relación entre el daño del ADN, la edad (años) y el tiempo de exposición (años en el oficio de pintor) se analizó mediante la prueba de correlación de Spearman, en tanto que la relación entre el daño en el ADN y el consumo de alcohol se analizó mediante la prueba U de Mann-Whitney.

El nivel crítico de rechazo de la hipótesis nula se estableció con valores de p de 0,05 o menos, y todos los datos se procesaron y analizaron con el programa estadístico SPSS™, versión 13 (SPSS Inc., Chicago, IL).

## Resultados

En el cuadro 1 se resumen las principales características demográficas de los grupos de estudio. La edad promedio en años y el consumo de alcohol en los dos grupos fueron similares, sin diferencias significativas (p=1,0).

**Cuadro 1:** Características demográficas de los grupos de estudio

Características	No expuestos n=62	Expuestos n=62	p
Edad (años)			
Media ± EE	40,35 ± 1,15	39,87 ± 1,11	0,76^a^
Rango (mínimo-máximo)	18-55	19-54	
Tiempo de exposición (años)			
Media ± EE	NA	19,10±1,054	NA
Rango (mínimo-máximo)	NA	5-38	NA
Uso de equipo de protección personal de las vías respiratorias, n (%)			
No usa	NA	39 (62,9)	NA
Usa	NA	23 (37,1)	NA
Consumo de alcohol, n (%)			
No consumen	15 (24,2)	15 (24,2)	1,000^b^
Consumo (entre bajo a moderado)	47 (75,8)	47 (75,8)	

EE: error estándar; NA: no aplica

^a^ Prueba t de Student

^b^ Prueba de ji al cuadrado

En la anamnesis los pintores manifestaron estar expuestos a una gran variedad de solventes como *thinner*, aguarrás, varsol, acetonas, gasolina, resinas (epóxido y poliuretano) y pinturas a base de solventes, entre otros. También, reportaron que su carga laboral variaba mucho (6 a 12 horas por día) y que el área de trabajo era poco ventilada.

Ninguno de los pintores usaba equipo de protección personal (guantes de silicona, traje protector y gafas) para evitar el contacto dérmico con los solventes y las pinturas. Solo el 37,1 % manifestó usar equipo de protección respiratoria, específicamente máscaras con filtros de carbón activado. No obstante, la mayoría de estos pintores reportaron que no cambiaban los filtros con frecuencia, por lo que esta variable no se tomó en cuenta para la estadística inferencial.

Con respecto al consumo de alcohol, el 75,8 % de los individuos reportó que era de bajo a moderado, y se catalogaron como consumidores sociales.

En el cuadro 2 se presentan los valores de la mediana y los rangos intercuartílicos del porcentaje estimado de ADN en la cola de los linfocitos de los individuos de ambos grupos de estudio para cada una de las categorías de daño analizadas. Los resultados evidenciaron una diferencia estadísticamente significativa (p≤0,005) en los porcentajes de ADN en la cola entre el grupo expuesto y el no expuesto en las tres categorías de daño evaluadas. Se observaron diferencias significativas (p<0,001) dentro de los grupos entre el daño oxidativo neto y el daño basal (valor de referencia). Por el contrario, no se registraron diferencias significativas (p*>*0,05) entre el daño neto por metilación y el daño basal.

**Cuadro 2:** Porcentaje de ADN en la cola estimado en linfocitos de individuos de los grupos de estudio según las categorías de daño en el ADN

Categorías de daño en el ADN	Porcentaje de ADN en la cola	
	No expuestos (n=62) Mediana; RIC	Expuestos (n=62) Mediana; RIC	p
Daño basal	7,81; 12,92	12,53; 20,89	<0,001^b^
Daño oxidativo neto	12,87; 53,83^c^	16,50; 46,49^c^	0,005^b^
Daño neto por metilación	7,73; 45,01	14,00; 37,77	<0,001^b^
p	<0,001^a^	<0,001a	

RIC: rango intercuartílico

a Significación estadística de las categorías de daño en el ADN (daño basal, daño oxidativo neto y daño neto por metilación) según la prueba de Kruskal-Wallis

b Significación estadística de cada categoría de daño mediante la prueba U de Mann-Whitney en los grupos de estudio (no expuesto y expuesto)

c Diferencias significativas (p<0,001) halladas mediante la prueba U de Mann-Whitney entre las categorías de daño oxidativo neto en el ADN con respecto al daño basal (sin enzimas) para cada grupo de estudio

Los resultados de la correlación de Spearman revelaron que no hubo diferencia estadísticamente significativa entre la edad y los valores de daño basal (p=0,535, r=0,056) y del daño neto por metilación del ADN (p=0,104, r=0,147), en tanto que sí se observó una asociación estadísticamente significativa entre el daño oxidativo neto y la edad (p<0,039, r=0,185).

En cuanto al tiempo de exposición (años) a los solventes orgánicos, no se observó una asociación estadísticamente significativa (p>0,05) entre esta variable y las tres categorías de daño en el ADN.

## Discusión

La exposición ocupacional a solventes orgánicos es un problema de salud pública a nivel mundial ^22^ y, según el Ministerio de la Protección Social de Colombia, también es de interés nacional ^23^. En las últimas décadas, en diversos estudios se ha evidenciado que el 80 % de todos los cánceres son causados por la exposición ambiental u ocupacional a agentes químicos, físicos o biológicos ^24^^,^^25^. Según la *International Agency for Research on Cancer* (IARC), hay suficiente información de que los solventes orgánicos que emplean los pintores de automóviles tienen potencial cancerígeno ^26^. En estudios de cohorte, se ha encontrado que los pintores tienen riesgo ocupacional de desarrollar cáncer de páncreas, pulmón y vejiga ^27^^-^^29^.

En cuanto a los efectos genotóxicos de estos solventes empleados en ese oficio, se ha observado que, en condiciones como las analizadas en el presente estudio, la exposición a solventes orgánicos y pinturas induce un incremento significativo (p=0,005) de la frecuencia de la ruptura de cadenas de ADN y de la presencia de purinas oxidadas (8-oxodG, FapydAde y FapydGua). Estos resultados permiten suponer que el daño del ADN se genera durante el metabolismo celular de los solventes orgánicos por el ingreso al organismo de los metales pesados contenidos en las pinturas, los cuales incrementan el estrés oxidativo y la producción de especies reactivas de oxígeno y de radicales libres en los individuos expuestos.

Estos datos respaldan la hipótesis de que el estrés oxidativo tendría un papel relevante en los efectos biológicos causados por los solventes orgánicos como causa principal o secundaria en la etiología de las enfermedades neurodegenerativas y el cáncer, entre otras ^28^.

Los pintores de automóviles en Cauca utilizan mezclas complejas de solventes orgánicos, principalmente thinner 7. Esta mezcla contiene diferentes compuestos orgánicos, entre los que se destacan el tolueno y el etilbenceno, los cuales tienen una alta afinidad con el sistema enzimático del metabolismo de xenobióticos; ya en el organismo y en las rutas metabólicas menores, el tolueno es biotransformado en metilhidroquinona y metilbenzoquinona, en tanto que el etilbenceno es convertido en etilhidroquinona y 4-etilcatecol. Durante los procesos de reducción-oxidación (redox), estas quinonas generan sobreproducción de especies reactivas de oxígeno, como el anión superóxido (O 2 ● ─) y los radicales hidroxilo ( ●OH) ^30^^-^^32^; estos, por ser fuertemente electrofílicos, interactúan con los centros nucleofílicos de las bases nitrogenadas y con los enlaces carbono-hidrógeno de las pentosas, e inducen ruptura de cadenas en el ADN y en los sitios sin purina ^33^, lo que se evidencia por el incremento del porcentaje de ADN en la cola en linfocitos de individuos expuestos.

Por otro lado, cuando lesiones como las 8-oxodG, FapydGua y FapydAde no se reparan eficientemente mediante la escisión de bases, desestabilizan la conformación electrónica del ADN, alteran las propiedades de apareamiento de las bases nitrogenadas y generan una mayor frecuencia de mutaciones del tipo de transversión GC→AT, AT→GC ^33^^-^^35^. El aumento en la frecuencia de este tipo de mutaciones en los genes que regulan procesos celulares (como la replicación del ADN, la progresión del ciclo celular y el control de los puntos de chequeo, la segregación de cromosomas y la reparación del ADN), conlleva la inestabilidad genómica característica de las células tumorales. Por lo tanto, los pintores de automóviles podrían considerarse una población en mayor riesgo de desarrollar cáncer u otras enfermedades degenerativas asociadas con alto grado de estrés oxidativo.

En este estudio el daño oxidativo del ADN concordó con el reportado por Martínez-Alfaro, *et al.*, quienes mediante la aplicación del ensayo cometa alcalino convencional y empleando la enzima Fpg detectaron un incremento significativo del daño del ADN de linfocitos de ratas expuestas a vapores de *thinner*, así como en las concentraciones de malondialdehído (producto de la peroxidación lipídica), y una reducción significativa en la expresión de glutatión (antioxidante no enzimático), biomarcadores ampliamente usados para determinar el estrés oxidativo ^6^.

En este estudio, el tratamiento con la enzima AlkA no reveló daño por metilación en los grupos de estudio. Es bien sabido que la mayoría de los agentes xenobióticos inducen daño oxidativo del ADN con mayor frecuencia y, en menor proporción, daño por metilación ^15^^,^^36^. Sin embargo, no se puede afirmar de forma concluyente que el material genético no haya sufrido este tipo de daño, puesto que los pintores de automóviles no solo están expuestos a productos aromáticos, como el tolueno y el etilbenceno, sino también a productos alifáticos metilados, como el 2,3-dimetilhexano (componente del *thinner*) o acetilados, que pueden ser transportados durante los procesos metabólicos e intervenidos por la acetil coenzima A, con lo que podrían liberar grupos metilo que se unen covalentemente al ADN.

De hecho, en un estudio de Hoyos, *et al*., se registró un incremento significativo de la metilación de regiones promotoras de genes específicos (GSTP1, p16(INK4a) y APC) de células epiteliales de la vejiga en pintores de automóviles, comparados con los individuos no expuestos ^37^. En este estudio, también se observó que, entre los individuos expuestos, aquellos con más años de exposición presentaban un incremento en la frecuencia de metilación.

Teniendo en cuenta que son relativamente pocos los estudios en los que se han evaluado los efectos de la exposición a solventes orgánicos y pinturas en la metilación del ADN, sería conveniente seguir investigando los mecanismos de este tipo de daño en individuos expuestos a solventes orgánicos y pinturas, ya que la hipermetilación del ADN puede tener un papel determinante en la aparición de tumores en los humanos por el aumento de la proliferación celular, la acumulación de metabolitos genotóxicos en las células y una mayor inestabilidad genómica y, por lo tanto, en el desarrollo de enfermedades como el cáncer.

Por otro lado, en el presente estudio, se encontró una asociación estadísticamente significativa (p<0,039) entre el daño oxidativo neto y la edad. Según Moller, el daño del ADN aumenta con la edad, lo que es entendible dado que, a mayor edad, menor es la eficiencia de la reparación del ADN ^38^. En un estudio de Mladinic, *et al.,* en una población saludable no expuesta a agentes químicos, se reportó un incremento significativo del daño del ADN de linfocitos de individuos ancianos (69,3 ± 3,7 años) con respecto a individuos jóvenes (38,9 ± 8,8 años), lo que evidencia la influencia positiva de la edad en los resultados obtenidos mediante el ensayo cometa ^39^. Por el contrario, no se encontró una asociación estadísticamente significativa entre la edad de los participantes del estudio y el daño basal (sin enzimas) (p=0,535) y el daño neto causado por metilación (p=0,104) en el ADN.

Estos resultados concuerdan con los obtenidos en otros estudios, en los cuales no se encontró una asociación significativa entre la edad y el daño en el ADN inducido por los solventes orgánicos ^40^^-^^42^, aunque también hay otros estudios de biomonitoreo en poblaciones expuestas ocupacionalmente a solventes orgánicos que indican que el envejecimiento es un factor que influye en la expresión del daño en el ADN estimado mediante el ensayo cometa ^2^^,^^3^. Por ello, la asociación positiva entre la edad y el daño en el ADN cuantificado mediante dicho ensayo es controversial y aún está en discusión.

Es importante mencionar que la mayoría de los estudios de biomonitoreo genético mediante el ensayo cometa convencional, no han analizado la influencia de la edad en la expresión del daño del ADN y se han limitado exclusivamente a describir esta variable en la información demográfica de la población objeto de estudio (estadística descriptiva). Por ello, es necesario que en futuros estudios se incluyan grupos numerosos de individuos de todas las edades, con el fin de establecer el efecto de la edad en la expresión del daño del ADN evaluado mediante el ensayo cometa.

En cuanto al tiempo de exposición, no se encontró una asociación significativa (p>0,05) entre este factor (determinado en años) y los diferentes tipos de daño del ADN (oxidativo y por metilación) en los linfocitos de los pintores de automóviles, resultados que coinciden con los obtenidos en algunos estudios consultados ^40^^,^^43^, aunque en otros se ha señalado que, cuanto mayor es el tiempo de exposición ocupacional a solventes orgánicos y pinturas, mayor es la expresión acumulada del daño en el ADN estimado mediante el ensayo cometa ^2^^,^^42^^,^^44^^).^

Por lo tanto, los resultados relacionados con la variable del tiempo de exposición son controversiales y se requiere de un mayor análisis en estudios de biomonitoreo genético para obtener información más concluyente sobre este tipo de asociación. Otro aspecto importante con respecto al análisis de esta variable es que, al tratarse de una población de pintores del sector informal de la industria, no hay certeza sobre el tiempo que llevaban dedicados de forma continua al oficio. Por ello, la información recolectada no permitió determinar los grupos o los tiempos de exposición de forma exacta para establecer relaciones directas con el tiempo de exposición crónica.

Una de las principales razones para el uso del ensayo cometa en este estudio fue la coherencia encontrada entre el incremento en los valores del daño del ADN en dicho ensayo y el incremento de la frecuencia de daños cromosómicos expresados en las células, como los micronúcleos y las alteraciones cromosómicas, los cuales son biomarcadores validados internacionalmente como predictores de riesgo de cáncer ^45^^,^^46^ en poblaciones expuestas a solventes orgánicos ^42^^,^^47^^-^^49^. Por lo tanto, el ensayo cometa de gran eficiencia puede considerarse una herramienta útil para los programas de vigilancia epidemiológica ocupacional, y para identificar y reducir los riesgos de la exposición ocupacional a solventes orgánicos y pinturas.

Por último, en este estudio se observó una asociación entre la exposición a mezclas complejas de solventes orgánicos y pinturas, y el daño oxidativo del ADN de linfocitos de sangre periférica. Además, el aumento del grado de daño oxidativo en el grupo expuesto podría sugerir que en estos individuos la reacción a la reparación del ADN no es tan eficiente como en los individuos del grupo no expuesto, específicamente mediante el mecanismo de escisión de bases, que permite reparar lesiones producidas por la oxidación de bases como las 8-oxodG, FapydGua y FapydAde.

Estos resultados alertan sobre el potencial genotóxico de los solventes orgánicos y las pinturas y el posible riesgo laboral de los pintores de desarrollar enfermedades crónicas como el cáncer y las neurodegenerativas. Asimismo, debe hacerse un llamado para que se diseñen estrategias de vigilancia epidemiológica ocupacional que permitan medir y mitigar los efectos adversos de dicha exposición, puesto que la eliminación del riesgo es poco probable en este sector informal de la industria. Sería conveniente, igualmente, motivar el desarrollo de actividades de capacitación para concientizar a los trabajadores sobre los riesgos de la exposición a agentes genotóxicos y la necesidad de cumplir con las mínimas medidas de protección personal en los espacios laborales y de adoptar hábitos saludables (autocuidado).

Aunque en este estudio se pudo recabar información preliminar sobre la interacción entre los genes y el ambiente en la población de pintores de automóviles, los resultados deben validarse en estudios prospectivos que proporcionen resultados más sólidos sobre dicha interacción.

En futuros estudios, también debería considerarse el aumento del tamaño de la muestra y hacer un muestreo probabilístico. Asimismo, deberán emplearse ‘biomarcadores’ de la vulnerabilidad individual, por ejemplo, la detección de polimorfismos en los genes del metabolismo o la reparación del ADN, que permitan una mejor comprensión de los mecanismos del daño y de la eficiencia de su reparación. También, deben establecerse los grados de exposición ambiental, como una buena alternativa para determinar el grado y el tipo de agentes genotóxicos a los que están expuestos los pintores.

Sin embargo, los muestreos en el área de trabajo no siempre resultan representativos de la exposición en esta población debido a la intermitencia de los trabajadores en el oficio y el lugar de trabajo, por lo que sería conveniente analizar marcadores de exposición biológica en sangre u orina, así como biomarcadores del efecto.
